# Lower energy intake associated with higher risk of cardiovascular mortality in chronic kidney disease patients on a low-protein diets

**DOI:** 10.1186/s12937-024-00980-y

**Published:** 2024-07-15

**Authors:** Yao Liu, Fei Deng, Ping Zhou, Cong Peng, ChunPeng Xie, Wuyu Gao, Qianyu Yang, Tingyu Wu, Xiang Xiao

**Affiliations:** 1 Department of Nephrology, The first affiliated hospital of Chengdu Medical college，School of Clinical Medicine, Chengdu Medical College, No. 278, Middle Section of Baoguang Avenue, Xindu District, Chengdu, Sichuan 610500 China; 2 School of Laboratory Medicine, Chengdu Medical College, Chengdu, 610500 China; 3 Department of Nephrology, Jinniu Hospital, Sichuan Provincial People’s Hospital, Jinniu People’s Hospital, Chengdu, 610072 China; 4 Department of Clinical Medicine, Chengdu Medical College, Chengdu, 610500 China

**Keywords:** Chronic kidney disease, Malnutrition, Mortality risk, Epidemiology and outcomes, Risk factors

## Abstract

**Objective:**

An increasing number of studies shown that inadequate energy intake causes an increase in adverse incidents in chronic kidney disease (CKD) patients on low-protein diets (LPD). The study aimed to investigate the relationship between energy intake and cardiovascular mortality in CKD patients on a LPD.

**Methods:**

This was a cross-sectional study, a total of 4264 CKD patients were enrolled from the NHANES database between 2009 and 2018. Restricted cubic spline plots and Cox regression analysis were used to analyze the association between energy intake and cardiovascular mortality in CKD patients on a LPD. Additionally, a nomogram was constructed to estimate cardiovascular survival in CKD patients on a LPD.

**Results:**

Among CKD patients on a LPD in the United States, 90.05% had an energy intake of less than 25 kcal/kg/day, compared to 36.94% in CKD patients on a non-LPD. Energy intake and cardiovascular mortality showed a linear relationship in CKD patients on a LPD, while a ‘U-shaped’ relationship was observed in CKD patients on a non-LPD. Multifactorial Cox regression models revealed that for Per-standard deviation (Per-SD) decrement in energy intake, the risk of cardiovascular mortality increased by 41% (HR: 1.41, 95% CI: 1.12, 1.77; *P* = 0.004) in CKD patients on a LPD. The concordance index of the nomogram was 0.79 (95% CI, 0.75, 0.83).

**Conclusion:**

CKD patients, especially those on a LPD, have significantly inadequate energy intake. Lower energy intake is associated with higher cardiovascular mortality in CKD patients on a LPD.

**Supplementary Information:**

The online version contains supplementary material available at 10.1186/s12937-024-00980-y.

## Introduction

Endemic with high morbidity and mortality, Chronic Kidney Disease (CKD) imposes a substantial global health and economic burden. The Global Burden of Disease Study 2017 estimates a global prevalence of 9.1%, with CKD-related mortality escalating by 41.5% between 1990 and 2017, ranking it the 12th leading global cause of mortality [1, 2]. Furthermore, CKD-related end-stage renal disease (ESRD) accounts for 2–3% of total healthcare expenditures in developed nations, underscoring its significant financial impact [3].

As a cornerstone of Medical Nutrition Therapy (MNT) for CKD management, a low-protein diet (LPD), typically curtailed to 0.6–0.8 g/kg/day [4–6], has been empirically validated to ameliorate albuminuria and decelerate the deterioration of the glomerular filtration rate (GFR) [7, 8]. Consequently, guidelines endorse its adoption in treating patients with this affliction [9–11].

The conundrum resides in harmonizing the renoprotective benefits of a LPD with the assurance of sufficient nutritional consumption to counteract malnutrition risks. Protein-energy wasting (PEW), a pervasive concern in CKD patients, especially those undergoing maintenance dialysis is primarily attributed to deficient dietary protein and energy intake [12]. Observational studies have documented a substantial PEW prevalence in CKD patients, oscillating between 40 and 70% [13–15]. This condition precipitates a compromised immune system, muscle atrophy, anemia, and additional complications, collectively escalating the mortality risk in CKD patients [16, 17]. Consequently, notwithstanding the advantages of LPD in decelerating the GFR decline in CKD, burgeoning evidence implies that this dietary regimen can exacerbate PEW, predominantly due to insufficient protein and energy intake [18, 19].

The inherent drawback of LPD, notably its propensity to induce deficient energy consumption, especially in those patient adhering to VLPD, further exacerbates the task of sustaining sufficient energy intake [4]. To counteract PEW risk in CKD patients on a LPD, augmenting energy intake is vital, given its potential to diminish PEW incidence and subsequent mortality. Nevertheless, there is a conspicuous absence of research probing the current state of energy consumption or the influence of energy intake on patient prognosis in CKD patients. Therefore, this study will serve to understand the current dietary consumption status and refine our prevailing dietary regimen in CKD patients, and explore the relationship between energy intake and cardiovascular mortality in CKD patients on a LPD.

## Materials and methods

### Study design and individuals

The National Health and Nutrition Examination Survey (NHANES) is an ongoing study that collects nutrition and health-related data from adults and children in the United States. The survey utilizes a stratified, multi-stage probability design to obtain a representative sample of the U.S. population. We utilized continuous data from NHANES 2009–2018, which provided information on total nutrient intake. Initially, as the COVID-19 pandemic originated in 2019, it has had a profound impact on the occurrence, treatment, and lifestyle management of many diseases. Therefore, we have decided to set the study’s cut-off date at 2018. Furthermore, in order to increase the sample size and enhance the reliability of our conclusions, we have established a time span of 10 years, with the study’s starting point set at 2009. Additionally, if the time span were earlier, significant differences in new treatment technologies and concepts might exist, potentially leading to some degree of heterogeneity. Hence, we have ultimately confined the study period to the years 2009 to 2018. Specifically, we extracted data on total protein, carbohydrate, fat, and energy intake on the first day of participation for individuals aged 20 and above [20]. Mortality data related to the participants were obtained from the National Death Index (NDI) database of the CDC, up until December 31, 2019. All data used in this study are publicly available at https://www.cdc.gov/nchs.

CKD is defined as abnormalities of kidney structure or function, present for > 3 months, with implications for health. The diagnostic criteria for CKD were 1) markers of kidney damage (one or more): (a) albuminuria (albumin-creatinine ratio (ACR) ≥ 30 mg/g; albumin excretion rate ≥ 30 mg/day), (b) urine sediment abnormalities, (c) electrolyte and other abnormalities due to tubular disorders, (d) abnormalities detected by histology, (e) structural abnormalities detected by imaging, (f) history of kidney transplantation; 2) decreased estimating glomerular filtration rate (eGFR), eGFR < 60 ml/min/1.73 m^2^; meets (1) or / and (2) [9, 21]. Exclusion criteria were: (1) age < 20 years, (2) pregnant, (3) missing information on dietary intake on the first day, (4) missing weight data, (5) missing mortality and weighing information, (6) missing survival information, (7) hemodialysis or peritoneal dialysis.

Some additional definitions for smoking, alcohol consumption, hypertension, diabetes, hyperlipidemia, low-carbohydrate diet, low-fat diet, and cardiovascular disease can be found in Supplementary Table 1. LPD was defined as a protein intake of less than 0.8 g/kg/day, and a VLPD was defined as a protein intake of less than 0.6 g/kg/day. Low-fat diets are considered as food where 30% or less of the calories come from fat [22]. Energy intake is categorized into three levels: <25 kcal/kg/day, 25-35 kcal/kg/day, and ≥ 35 kcal/kg/day. Energy intake inadequacy is defined as less than 25 kcal/kg/day [23]. Resting energy expenditure (REE) was calculated using the energy equation [24]. Comprehensive laboratory analyses, including ACR, eGFR, and serum albumin at baseline, were performed. The eGFR was calculated using for the CKD-EPI. Baseline corresponds to the NHANSE study’s population enrollment time point.

### Statistical analysis

In all analyses, we accounted for weighted samples and deliberate stratification and clustering to extrapolate estimates for the general U.S. populace, adhering to CDC guidelines. Continuous and categorical variables were articulated as weighted means (standard errors (SE)) and count (weighted percentages) respectively. We utilized weighted t-tests and chi-square tests to discern differences between groups for continuous and categorical variables correspondingly. To scrutinize the dose-response correlation between energy intake and cardiovascular mortality, we conducted a restricted cubic spline (RCS) with three knots. Individuals were segregated into three groups based on energy intake using the triple quantile method. We then examined the correlation between energy intake and cardiovascular mortality risk in individuals with CKD on a LPD using a weighted multivariate Cox regression model, assessing result consistency through stratified analysis. Inconsistent results in stratified analyses warranted subgroup analyses for further clarity on the more significant correlations. To ensure the accuracy of the results, a sensitivity analysis of the data was conducted. Firstly, after deleting the outliers values or performing multiple imputations, a multivariate Cox regression analysis was performed. Secondly, the patients were grouped respectively according to the different energy intake levels using the dichotomous method and the quartile method, and then a multivariate Cox regression analysis was performed. Thirdly, in order to judge the possible reduction in energy intake due to dietary intake problems caused by specific diseases that may occur, the incidences of diseases such as Digestive System Tumors (including colorectal cancer, esophageal cancer, liver cancer, and gallbladder cancer), COPD, and depressive disorders were compared among patients with different energy intakes. Finally, the patients with diseases that may affect the dietary intake were deleted, and then a multivariate Cox regression analysis was conducted. Nomograms for complex sampling, constructed from weighted multivariate Cox regression models, estimated cardiovascular survival probabilities at 3 and 5 years. Concordance indices and calibration curves estimated the concordance between actual observations and nomogram-predicted survival probabilities. Missing values were addressed via multiple interpolation. All statistical tests were processed using R 4.2.2. A two-sided *P* < 0.05 was deemed statistically significant.

## Results

### Baseline characteristics and status of dietary intake in patients with CKD

**Fig. 1:**
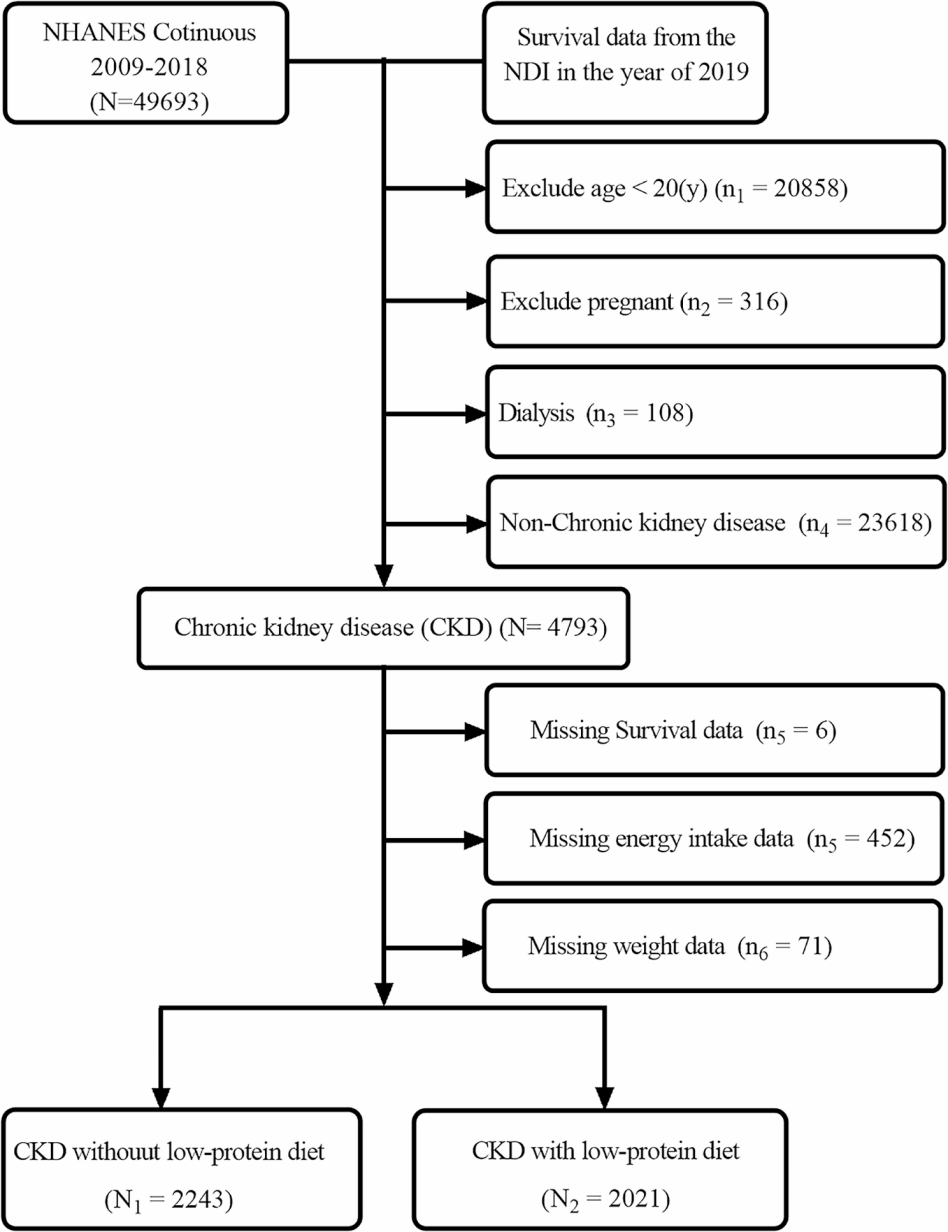
Flowchart of included individuals in this study

The study encompassed data from 49,693 individuals within the NHANES registry (2009–2018). Post-screening, 4,264 CKD individuals fitting our criteria were analyzed (Fig. 1). Of these, 47.4% (2021/4264) adhered to a LPD. The average protein consumption for CKD patients was 0.92 (0.01) g/kg/day, with LPD and non-LPD patients consuming 0.54 (0.01) g/kg/day and 1.24 (0.01) g/kg/day respectively. The proportion of LPD adherence stood at 46.43 (0.02), with 26.67 (0.01) following a VLPD. CKD patients exhibited a low-carbohydrate diet ratio of 2.27 (0.00), a low-fat plus low-carbohydrate diet ratio of 0.28 (0.01), and a low-protein plus low-fat plus low-carbohydrate intake ratio of 0.23 (0.00). LPD patients demonstrated a significantly higher prevalence of low-fat diets (37.38 vs. 25.06, *P* < 0.001) and low-fat plus low-carbohydrate diets (0.49 vs. 0.10, *P* = 0.046) compared to non-LPD patients.

CKD patients’ mean energy intake was 24.09 (0.26) kcal/kg/day. LPD patients consumed 16.74 (0.20) kcal/kg/day, significantly less than non-LPD patients’ 30.47 (0.37) kcal/kg/day (*P* < 0.001). Overall, 61.60 (0.02)% consumed less than 25 kcal/kg/day, with 90.05 (0.93)% of LPD patients and 36.94 (1.59)% of non-LPD patients. The proportion of LPD patients consuming less than 25 kcal/kg/day was significantly higher (*P* < 0.001). 31.57(0.01)% of patients’ intake was lower than the REE estimate, significantly more so in LPD patients than those non-LPD (56.59(1.67) vs. 9.89(0.82), *P* < 0.001). LPD patients were older, predominantly female, had a higher BMI, smoked more, had worse eGFR, lower serum albumin, and higher rates of hypertension, diabetes, anemia, and hyperlipidemia (*P* < 0.05) (Table 1).

**Table 1:** Baseline clinical features of enrolled CKD patients

Variable	Total (*N* = 4264)	Non-LPD (≥ 0.8 g/kg/day) (*n* = 2243)	LPD (< 0.8 g/kg/day) (*n* = 2021)	*P*-value
Age (years)	61.04(0.36)	60.31(0.49)	61.88(0.51)	0.02
Sex (%)				0.005
Female	56.67(0.02)	53.80(1.66)	59.98(1.52)	
Male	43.33(0.02)	46.20(1.66)	40.02(1.52)	
Ethnicity (%)				< 0.001
Mexican American	7.22(0.01)	8.07(1.10)	6.23(0.81)	
Non-Hispanic Black	12.36(0.01)	10.25(1.07)	14.78(1.26)	
Non-Hispanic White	68.24(0.03)	68.17(1.95)	68.33(1.82)	
Other Hispanic	4.81(0.00)	5.43(0.67)	4.09(0.52)	
Other ethnicity - Including Multi-Racial	7.38(0.01)	8.07(0.86)	6.57(0.82)	
BMI (kg/m^2^)				< 0.001
<30	23.00(0.01)	32.36(1.34)	12.19(1.04)	
≥30	77.00(0.02)	67.64(1.34)	87.81(1.04)	
Energy intake (kcal/kg/day)	24.09(0.26)	30.47(0.37)	16.74(0.20)	< 0.001
Energy intake (kcal/kg/day)				< 0.001
< 25	61.60(0.02)	36.94(1.59)	90.05(0.93)	
25–35	23.94(0.01)	36.90(1.60)	8.98(0.96)	
≥ 35	14.46(0.01)	26.15(1.33)	0.97(0.38)	
Energy intake greater than the equation-based REE (%)				< 0.001
No	31.57(0.01)	9.89(0.82)	56.59(1.67)	
Yes	68.43(0.02)	90.11(0.82)	43.41(1.67)	
Protein intake (g/kg/day)	0.92(0.01)	1.24(0.01)	0.54(0.01)	< 0.001
LPD (g/kg/day) (%)				
<0.8	46.43(0.02)	0.00(0.00)	100.00(0.00)	
≥0.8	53.57(0.02)	100.00(0.00)	0.00(0.00)	
Very low protein diet (g/kg/day) (%)				< 0.001
≥0.6	73.33(0.03)	100.00(0.00)	42.67(1.77)	
<0.6	26.67(0.01)	0.00(0.00)	57.33(1.77)	
Carbohydrate	2.90(0.03)	3.54(0.05)	2.17(0.03)	< 0.001
Low-carbohydrate diet (%)				0.37
No	97.73(0.03)	98.07(0.45)	97.35(0.63)	
Yes	2.27(0.00)	1.93(0.45)	2.65(0.63)	
Fat (g/kg/day)	0.93(0.01)	1.20(0.02)	0.62(0.01)	< 0.001
Low-fat diet (%)				< 0.001
No	69.22(0.02)	74.94(1.21)	62.62(1.65)	
Yes	30.78(0.01)	25.06(1.21)	37.38(1.65)	
Low-fat and carbohydrate diet (%)				< 0.001
No	99.72(0.03)	99.90(0.07)	99.51(0.26)	
Yes	0.28(0.01)	0.10(0.07)	0.49(0.26)	
Low-LPD and fat and carbohydrate diet (%)				0.047
No	99.77(0.03)	100.00(0.00)	99.51(0.26)	
Yes	0.23(0.00)	0.00(0.00)	0.49(0.26)	
Alcohol use (%)				0.19
No	15.31(0.01)	14.46(0.92)	16.28(1.16)	
Yes	84.69(0.03)	85.54(0.92)	83.72(1.16)	
Smoke (%)				0.01
No	51.33(0.02)	54.30(1.66)	47.91(1.71)	
Yes	48.67(0.02)	45.70(1.66)	52.09(1.71)	
ACR (mg/g)				0.19
<30	33.30(0.02)	31.84(1.37)	34.98(1.66)	
30–300	56.75(0.02)	58.38(1.43)	54.87(1.68)	
≥300	9.95(0.01)	9.78(0.89)	10.15(0.82)	
e-GFR (ml/min/1.73m^2^)				< 0.001
≥90	30.63(0.01)	34.39(1.35)	26.28(1.37)	
60–89	21.41(0.01)	21.45(1.31)	21.37(1.36)	
30–59	44.60(0.02)	41.84(1.41)	47.78(1.70)	
15–29	3.00(0.00)	2.01(0.29)	4.15(0.52)	
< 15	0.36(0.00)	0.30(0.11)	0.42(0.14)	
Serum albumin (g/L)				0.01
≥35	97.52(0.03)	98.29(0.30)	96.63(0.61)	
<35	2.48(0.00)	1.71(0.30)	3.37(0.61)	
Hypertension (%)				< 0.001
No	31.90(0.02)	36.27(1.54)	26.86(1.63)	
Yes	68.10(0.02)	63.73(1.54)	73.14(1.63)	
Diabetes mellitus (%)				< 0.001
No	64.62(0.02)	71.08(1.45)	57.17(1.53)	
Yes	35.38(0.01)	28.92(1.45)	42.83(1.53)	
Anemia (%)				< 0.001
No	85.22(0.03)	88.04(0.91)	81.96(1.09)	
Yes	14.78(0.01)	11.96(0.91)	18.04(1.09)	
Hyperlipidemia (%)				< 0.001
No	18.37(0.01)	21.77(1.27)	14.44(1.24)	
Yes	81.63(0.03)	78.23(1.27)	85.56(1.24)	

LPD, low protein diet; BMI, body mass index; REE, resting energy expenditure; ACR, albumin-creatinine ratio; e-GFR, estimated glomerular filtration rate; CKD, chronic kidney disease

**Fig. 2:**
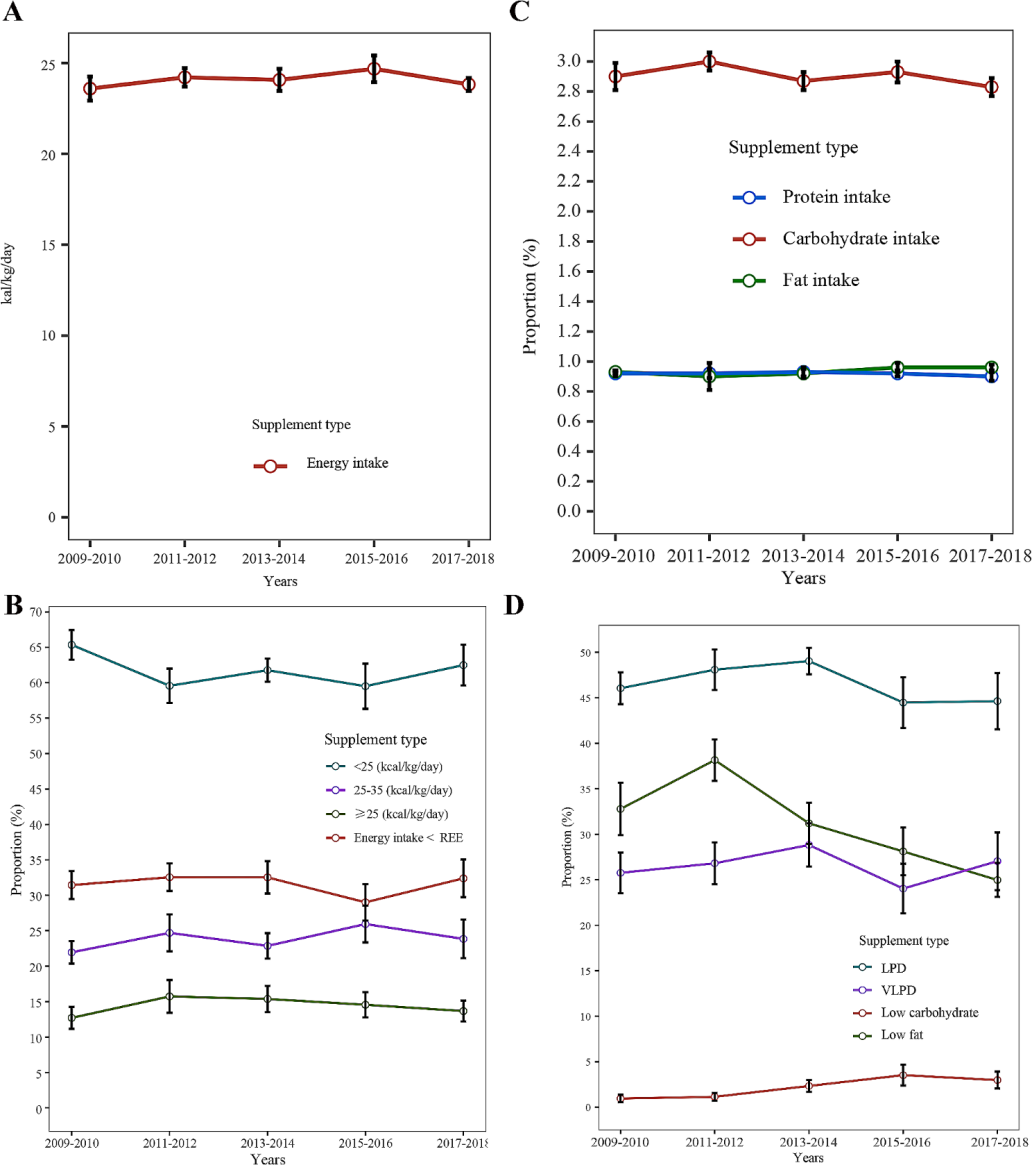
Status of nutritional intake in CKD patients with changes over the years: **(A)** energy intake, **(B)** the proportion of patients with different levels of energy intake, **(C)** protein, carbohydrate, and fat intake, **(D)** the proportion of patients with LPD, VLPD, low carbohydrate diet, low fat diet. LPD, low protein diet; VLPD, very low protein diet; CKD, chronic kidney disease

From 2009 to 2018, no notable alterations were discerned in the consumption of energy, protein, carbohydrates, and fats. In a similar vein, the percentage of CKD patients adhering to LPD, VLPD, maintaining an energy intake beneath REE, complying with low carbon diets, and sustaining an energy intake below 25 kcal/kg/day remained unvaried over time. Nonetheless, a significant downward trajectory was noted in CKD patients adhering to low-fat diets, low-carb plus low-fat diets, and low-protein-carbon-fat dietary intake patterns post-2013 (*P* < 0.05). (Supplementary-Table 2; Fig. 2).

### A dose-response association between energy intake and cardiovascular mortality in CKD patients

**Fig. 3:**
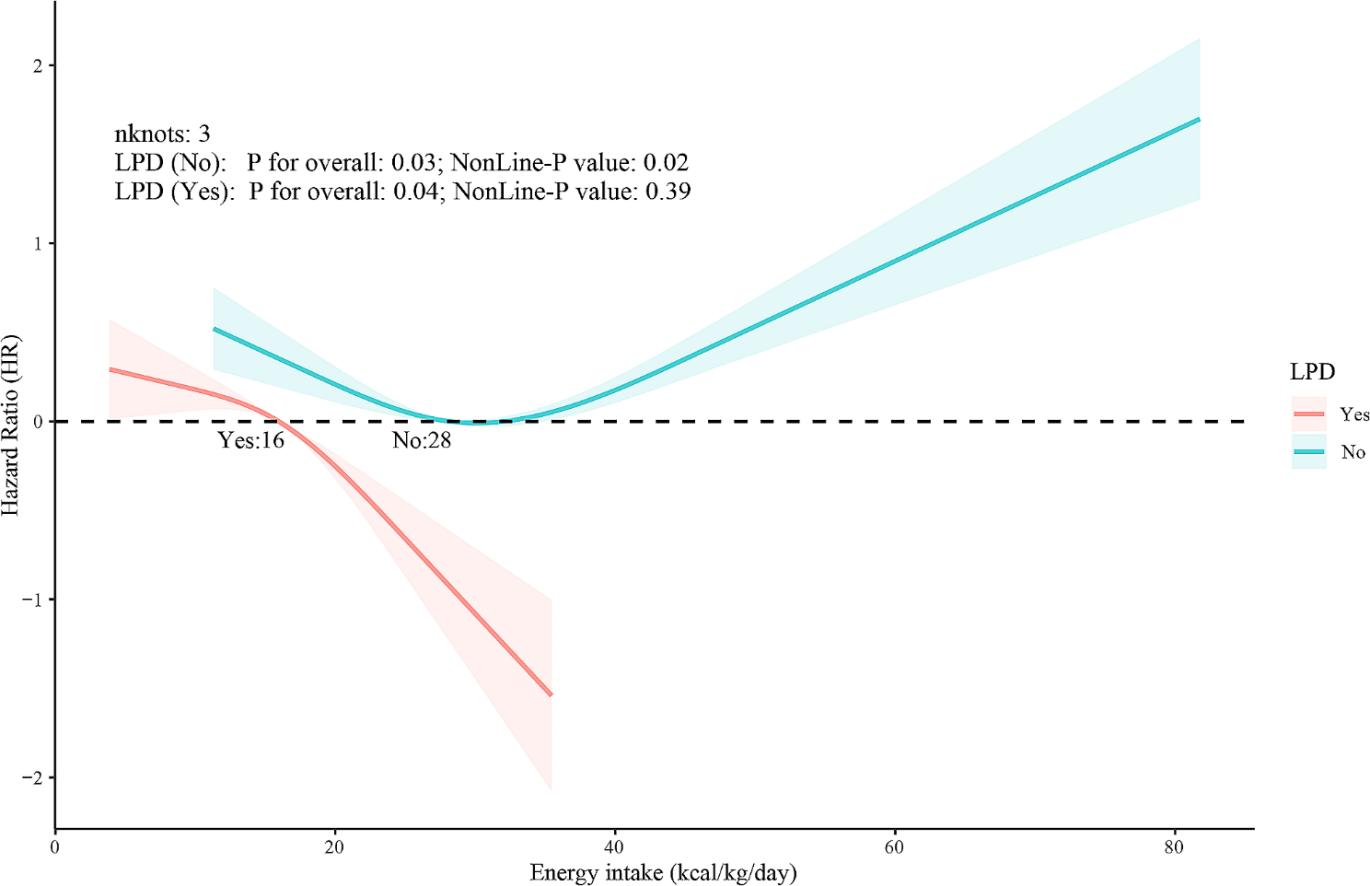
Association between energy intake and the risk of cardiovascular mortality in individuals with CKD by different protein intake based on restricted cubic spline plot

Given the notable energy shortfall in CKD patients, particularly those on a LPD, we divided the patients into two unique cohorts: those on a LPD and those not. We subsequently scrutinized the correlation between energy consumption and cardiovascular mortality risk for each group independently. Utilizing RCS for graphical depiction of this association, the findings revealed a linear relationship between energy intake and cardiovascular mortality risk among CKD patients on a LPD, with a decrease in mortality risk as energy intake increased (Non-Line *P* = 0.39). Conversely, for CKD patients not on a LPD, energy intake exhibited a ‘U-shaped’ pattern with respect to cardiovascular mortality risk, with a threshold value of 28 kcal/kg/d (Non-Line *P* value = 0.02) (Fig. 3).

### Risk assessment for the association between energy intake and cardiovascular mortality in patients with CKD on a LPD

**Fig. 4:**
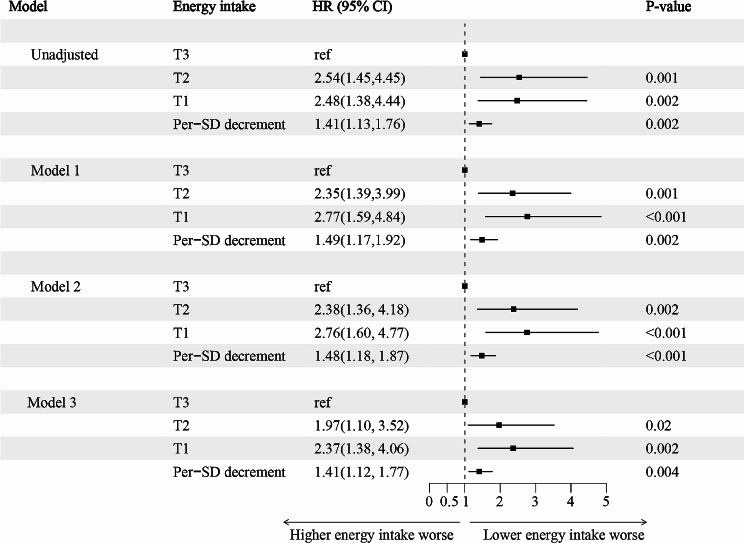
Associations between energy intake and the risk of cardiovascular mortality in individuals with CKD on a LPD. Model 1 adjusted for baseline age, sex (‘Male’, ‘Female’), ethnicity, BMI (‘<30’, ‘≥30’), alcohol use (‘No’, ‘Yes’), smoking (‘No’, ‘Yes’); Model 2 adjusted for covariates in model 1 plus e-GFR, ACR (‘<30’, ‘30–300’, ‘≥30’); Model 3 adjusted for covariates in model 2 plus serum albumin (‘<35’, ‘≥35’), hypertension (‘Yes’ or ‘No’), anemia (‘Yes’ or ‘No’), diabetes (‘Yes’ or ‘No’), hyperlipidemia (‘Yes’ or ‘No’), RAASi use (‘Yes’ or ‘No’); HR, hazard ratio; CI, confidence interval; BMI, body mass index; ACR, albumin-creatinine ratio; e-GFR, estimated glomerular filtration rate; RAASi, renin-angiotensin-aldosterone system inhibitor; CKD, chronic kidney disease

In our study of CKD patients on a LPD, we investigated if energy intake could predict cardiovascular mortality, given the severe inadequacy and its linear correlation with mortality risk. We divided 2021 patients into three tertiles based on energy intake, including 674 patients in the tertile 1 (≤ 13.46 kcal/kg/day), 673 patients in the tertile 2 (13.46–18.58 kcal/kg/day), and 674 patients in the tertile 3 (> 18.58 kcal/kg/day). Those with higher intake had lower BMI, higher eGFR, and fewer instances of hypertension and diabetes (*P* < 0.05) (Table 2). During follow-up, 133 patients experienced cardiovascular mortality, including 47 deaths in tertile 1, 52 deaths in tertile 2, and 34 deaths in tertile 3 (Table 2). Multifactorial Cox regression analysis revealed a significant association between lower energy intake and higher cardiovascular mortality risk, even after adjusting for various factors including age, sex, race, BMI, alcohol use, smoking, eGFR, ACR, serum albumin, hypertension, anemia, hyperlipidemia, diabetes, and RAASi use. Compared to tertile 3, the risk increased by 97% (HR: 1.97, 95% CI: 1.10, 3.52; *P* = 0.02) in tertile 2 and 137% (HR: 2.37, 95% CI: 1.38, 4.06; *P* = 0.003) in tertile 1. Per standard deviation (Per-SD) decrease in energy intake was linked with a 41% (HR: 1.41, 95% CI: 1.12, 1.77; *P* = 0.004) increased risk of cardiovascular mortality (Supplementary-Table 3 and 4; Fig. 4).

**Table 2:** Baseline clinical features of enrolled CKD patients on a LPD

Variable	Total (*N* = 2021, unweighted)	Tertile 1 (≤ 13.46 kcal/kg/day) (*n* = 674, unweighted)	Tertile 2 (13.46–18.58 kcal/kg/day) (*n* = 673, unweighted)	Tertile 3 (> 18.58 cal/kg/day) (*n* = 674, unweighted)	*P*-value
Energy intake (kcal/kg/day)	16.74(0.20)	10.15(0.13)	16.00(0.08)	23.72(0.26)	< 0.001
Age (years)	61.88(0.51)	61.51(0.86)	63.02(0.73)	61.13(1.02)	0.19
Sex (%)					0.47
Female	59.98(0.03)	61.57(2.52)	57.39(2.62)	60.98(2.70)	
Male	40.02(0.02)	38.43(2.52)	42.61(2.62)	39.02(2.70)	
Ethnicity (%)					0.55
Mexican American	6.23(0.01)	5.99(1.20)	6.32(1.13)	6.38(1.13)	
Non-Hispanic Black	14.78(0.01)	16.90(1.88)	14.11(1.38)	13.42(1.53)	
Non-Hispanic White	68.33(0.04)	67.88(2.36)	67.49(2.21)	69.56(2.62)	
Other Hispanic	4.09(0.00)	3.66(0.81)	4.96(0.91)	3.65(0.75)	
Other ethnicity - Including Multi-Racial	6.57(0.01)	5.56(0.98)	7.13(1.15)	6.99(1.17)	
BMI (kg/m^2^)					< 0.001
<30	23.00(0.01)	8.38(0.92)	17.20(1.29)	42.13(1.71)	
≥30	77.00(0.02)	91.62(0.92)	82.80(1.29)	57.87(1.71)	
Alcohol use (%)					0.06
No	16.28(0.01)	19.85(2.38)	13.01(1.71)	16.06(1.83)	
Yes	83.72(0.04)	80.15(2.38)	86.99(1.71)	83.94(1.83)	
Smoke (%)					0.45
No	47.91(0.02)	47.27(2.85)	45.72(3.04)	50.63(2.72)	
Yes	52.09(0.03)	52.73(2.85)	54.28(3.04)	49.37(2.72)	
ACR (mg/g)					0.09
<30	34.98(0.02)	32.75(2.79)	34.24(2.87)	37.81(2.52)	
30–300	54.87(0.02)	56.59(3.28)	52.38(3.01)	55.64(2.49)	
≥300	10.15(0.01)	10.66(1.85)	13.38(2.10)	6.56(1.15)	
e-GFR (ml/min/1.73m^2^) (%)					0.02
≥90	26.28(0.02)	21.84(2.22)	26.73(2.58)	30.07(2.75)	
60–89	21.37(0.02)	23.02(2.32)	22.81(2.39)	18.41(1.94)	
30–59	47.78(0.03)	48.20(2.87)	46.64(2.67)	48.48(2.66)	
15–29	4.15(0.01)	6.72(1.18)	3.20(0.75)	2.62(0.60)	
< 15	0.42(0.00)	0.22(0.11)	0.62(0.23)	0.41(0.30)	
Serum albumin (g/L)					0.64
<35	3.37(0.01)	3.76(0.84)	3.59(0.93)	2.79(0.87)	
≥35	96.63(0.04)	96.24(0.84)	96.41(0.93)	97.21(0.87)	
Hypertension (%)					0.01
No	26.86(0.02)	25.64(2.83)	21.54(2.34)	33.17(2.96)	
Yes	73.14(0.03)	74.36(2.83)	78.46(2.34)	66.83(2.96)	
Diabetes mellitus (%)					< 0.001
No	57.17(0.03)	49.55(2.84)	51.53(3.09)	69.87(2.33)	
Yes	42.83(0.02)	50.45(2.84)	48.47(3.09)	30.13(2.33)	
Anemia (%)					0.87
No	81.96(0.04)	81.39(1.88)	81.78(2.13)	82.69(1.52)	
Yes	18.04(0.01)	18.61(1.88)	18.22(2.13)	17.31(1.52)	
Hyperlipidemia (%)					0.08
No	14.44(0.01)	12.39(1.71)	13.34(1.80)	17.46(2.03)	
Yes	85.56(0.04)	87.61(1.71)	86.66(1.80)	82.54(2.03)	
RAASi					0.65
No	80.80(0.03)	80.26(2.38)	79.54(2.28)	82.54(2.24)	
Yes	19.20(0.01)	19.74(2.38)	20.46(2.28)	17.46(2.24)	
CVD					0.03
No	70.70(0.03)	70.88(2.38)	66.08(2.51)	74.99(2.10)	
Yes	29.30(0.02)	29.12(2.38)	33.92(2.51)	25.01(2.10)	
Cardiovascular mortality [%(n)] (unweighted)	6.6(133/2021)	6.9(47/674)	7.7(52/673)	5.0(34/674)	

LPD, low protein diet; BMI, body mass index; ACR, albumin-creatinine ratio; e-GFR, estimated glomerular filtration rate; RAASi, renin-angiotensin-aldosterone system inhibitor; CKD, chronic kidney disease; CVD, cardiovascular disease

### Subgroup analysis

**Fig. 5:**
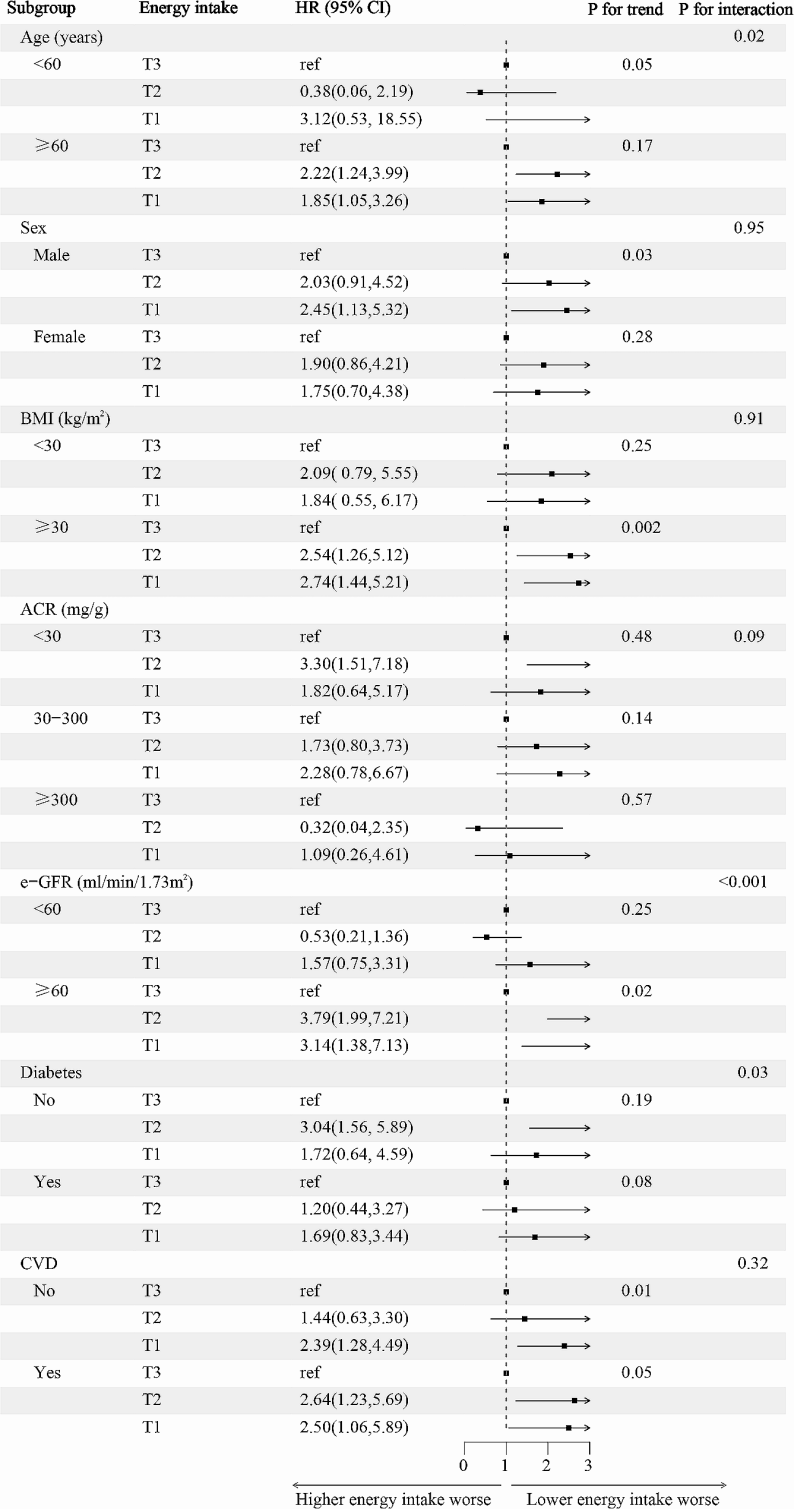
Stratified analysis of the effect of energy intake and the cardiovascular mortality in patients with CKD on a LPD. Adjusted for baseline age, sex, ethnicity, BMI, e-GFR, ACR, e-GFR; HR, hazard ratio; CI, confidence interval; ACR, albumin-creatinine ratio; e-GFR, estimated glomerular filtration rate; CKD, chronic kidney disease; CVD, Cardiovascular Disease

Stratified analyses scrutinized the correlation between energy consumption and cardiovascular mortality across diverse subgroups, revealing consistent associations across sex, BMI categories, ACR, and CVD history (Fig. 5; Supplementary-Table 5). However, inconsistencies arose across age, eGFR, and diabetes history. Further subgroup analyses, particularly for age, eGFR, and diabetes history, indicated that decreased energy intake correlated with increased cardiovascular mortality risk in younger, eGFR < 45 ml/min/1.73m^2^, and diabetes history in CKD patients on a LPD (Supplementary-Table 6)  .

### Sensitivity analysis

Upon the deletion of the extreme values of energy intake, the result of the multi-factor Cox regression analysis reveals that, in comparison with tertile 3, the risk increased by 129% (HR: 2.19, 95% CI: 1.21, 3.95; *P* = 0.01) in tertile 2 and by 81% (HR: 1.81, 95% CI: 1.08, 3.04; *P* = 0.03) in tertile 1. For Per-SD decrease in energy intake, there was an associated 33% (HR: 1.33, 95% CI: 1.09, 1.63; *P* = 0.005) increased risk of cardiovascular mortality (Supplementary-Table 7).

After multiple imputation for all variable missing values, the results of the multi-factor Cox regression analysis showed that, compared to tertile 3, the risk increased by 102% (HR: 2.02, 95% CI: 1.06, 3.86; *P* = 0.03) in tertile 2 and 125% (HR: 2.25, 95% CI: 1.33, 3.81; *P* = 0.003) in tertile 1. For Per-SD decrease in energy intake, it was associated with a 42% (HR: 1.42, 95% CI: 1.11, 1.81; *P* = 0.005) increased risk of cardiovascular mortality (Supplementary-Table 8).

Grouping was conducted according to the energy intake of patients by using the dichotomous method. The result of the multivariate Cox regression analysis indicated that, compared to Q2, the risk of cardiovascular mortality increased by 91% (HR: 1.91, 95% CI: 1.20, 3.05; *P* = 0.01) in Q1 (Supplementary-Table 9). Grouping was carried out according to the energy intake of patients by employing the quartile method. The result of the multivariate Cox regression analysis demonstrated that, compared to quartile 4, the risk increased by 130% (HR: 2.30, 95% CI: 1.30, 4.07; *P* = 0.004) in quartile 1 (Supplementary-Table 10).

Among CKD patients with different energy intakes, there is no significant difference in the incidence rates of diseases such as Digestive System Tumors (including colorectal cancer, esophageal cancer, liver cancer, and gallbladder cancer), COPD, and depressive disorders (All *P* > 0.05) (Supplementary-Table 11).

Exclude the patients with diseases that may affect dietary intake (Digestive System Tumors, COPD, and depressive disorders). The results of the multi-factor Cox regression show that, compared to tertile 3, the risk increased by 156% (HR: 1.56, 95% CI: 1.34, 4.91; *P* = 0.004) in tertile 1 (Supplementary-Table 12).

### Nomogram prediction of cardiovascular survival probability

**Fig. 6:**
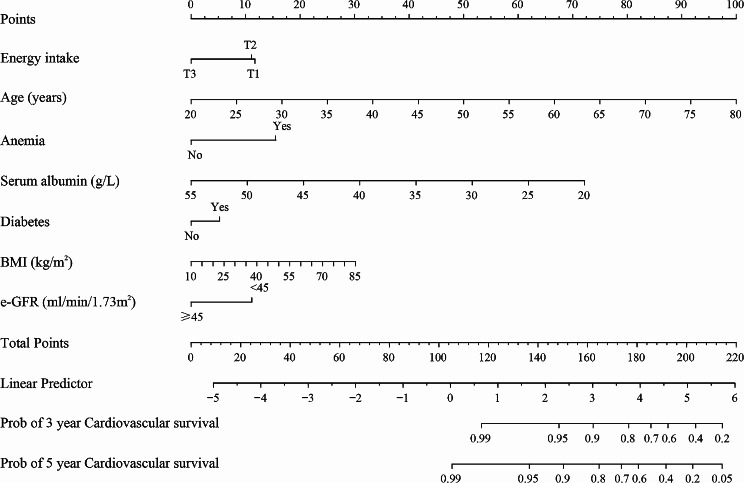
Nomograms predicted cardiovascular mortality. Prognostic nomogram to predict individual renal survival probability at 3, and 5 years in patients with CKD on a LPD. BMI, body mass index; e-GFR, estimated glomerular fltration rate

The nomogram amalgamates diverse autonomous risk elements, encompassing energy intake, age, anemia, serum albumin, BMI, eGFR, and diabetes, discerned through weighted multivariate Cox regression scrutiny. It proffers a rudimentary estimation method for prognosticating 3-, and 5-year cardiovascular survival likelihood in CKD patients on a LPD **(**Fig. 6**)**. For illustration, a CKD patient on a LPD, energy consumption = 15 kcal/kg/day (12.5 points), age = 65years (75 points), anemia = ‘Yes’ (16 points), serum albumin = 30 g/L (52.5 points), diabetes = ‘Yes’ (7.5 points), BMI = 40 kg/m^2^ (12.5 points), eGFR = 40 ml/min/1.73m^2^ (12.5 points), and cumulative total score 188.5 points which corresponded to the 3, and 5-year renal survival likelihood of approximately 65%, and 50%. The concordance index (C-index) of the nomogram was 0.79 (95% CI, 0.75, 0.83) which signifies its commendable discriminatory and predictive prowess. Moreover, the calibration curve displayed exceptional consistency between the nomogram-predicted cardiovascular survival likelihood and the actual observed outcome at 3 and 5 years (Supplementary -Fig. 1).

## Discussion

In our study, we explored the current energy intake status in patients with CKD, analyze the association between energy intake and cardiovascular mortality in patients with different protein intakes, and specifically focus on the association between different energy intakes and cardiovascular mortality in patients following a LPD. By sensitivity analysis, we obtained consistent results. Additionally, we constructed a simple estimation method of cardiovascular survival using a nomogram for CKD patients on a LPD.

The initial scholarly report on a LPD impact on chronic renal failure patients was published over half a century ago [25]. Subsequent studies have consistently affirmed LPD’s efficacy in decelerating CKD progression and reducing overall mortality [7, 8, 26], hence its inclusion in CKD’s MNT guidelines [9–11]. Yet, LPD compliance among CKD patients varies significantly, ranging from 9 to 72% [28–31], attributed to dietary habits and LPD’s inconsistent definitions. Our research revealed that the average protein intake of < 0.8 g/kg/day and < 0.6 g/kg/day in the US CKD population from 2009 to 2018 was 46.43 (0.02) and 26.67 (0.01) respectively. However, limited to the NHANES cohort, it only recorded the dietary status of patients who had just enrolled in the study, which may not reflect the overall LPD compliance. However, year-over-year analysis showed no significant difference, suggesting a close resemblance to the LPD proportion in US CKD patients. Additionally, we noted a declining trend in CKD patients on low-fat, low-carb plus low-fat, and low protein plus low carbon plus low fat diets post-2013.

The ISRNM-proposed PEW [14] concept in 2007 precipitates a compromised immune system, muscle atrophy, anemia, and additional complications, collectively escalating CKD patients’ mortality risk [16, 17], predominantly due to insufficient protein and energy intake [18, 19]. Given the LPD’s imperative for CKD patients to mitigate proteinuria and decelerate GFR deterioration, enhancing PEW through protein intake augmentation is impracticable. Hence, the sole alternative is to bolster PEW by escalating energy consumption. A prospective, randomized, open-label, controlled clinical trial involving 109 CKD patients demonstrated that adequate energy supplementation, combined with LPD, could deter PEW development and enhance LPD adherence [32]. However, the prevailing energy intake status among CKD patients remains uncertain. Given protein’s role as a primary energy source, CKD patients adhering to a LPD may be particularly vulnerable to insufficient energy intake [33], especially those limiting carbohydrate or fat consumption due to diabetes or cardiovascular disease. Studies have shown that energy intake is severely inadequate in hemodialysis and peritoneal dialysis patients [34, 35]. The NHANES data from 1999 to 2006 showed that total daily energy intake was also lower in the CKD population than in the non-CKD group [36].

Our investigation unveils a marked energy deficit in US CKD patients, particularly those on a LPD, with 61.6% of all and 90.0% of patients on a LPD falling below the guideline-recommended [23] minimum. Over 31.6% of CKD and 56.6% of patients on a LPD energy intake less than their REE. Despite not using indirect calorimetry, equation-based estimates were highly accurate [23]. This highlights a significant energy deficiency requiring urgent attention and action.

Refined dietary practices, particularly the reduction of energy intake, have proven beneficial for health and longevity across multiple organisms. Yet, it’s vital to underscore such reduction should be moderate, avoiding malnutrition. In chronic heart failure patients, diminished energy intake correlates with increased mortality rates and heart failure-related hospital admissions [37]. This could stem from the negative energy balance due to chronic energy deficiency, triggering protein degradation, sarcopenia, and cardiac cachexia. In a prospective study by Berbel MN et al. [38], low energy intake was linked to a heightened mortality risk in acute kidney injury patients. Regrettably, few research explores the correlation between energy intake and cardiovascular mortality in CKD patients.

In this study, we explored the correlation between energy intake and cardiovascular mortality in CKD patients on a LPD or non-LPD. The results unveiled a compelling pattern: a linear association between energy intake and cardiovascular mortality was observed in CKD patients on a LPD, while a ‘U-shaped’ correlation was found in those on a non-LPD. This implies a potential epidemiological discrepancy in these two types of patient. The observed difference may be attributed to the significantly lower average energy intake in patients on a LPD, obscuring the cardiovascular risks associated with higher intake. Conversely, non-LPD patients with excessive energy intake displayed an escalating risk of cardiovascular mortality as energy intake exceeded the cutoff value of 28 kcal/kg/day. Intriguingly, even below this cutoff value, a similar increasing risk was noted in patients on a non-LPD, mirroring the trend in patients on a LPD.

Given the grave concern of insufficient energy intake in CKD patients on a LPD, and its peculiar link to cardiovascular mortality, we undertook a thorough exploration of this correlation. Our definitive results reveal a 41% escalation in cardiovascular mortality risk per-SD reduction in energy intake, following a multifactorial Cox regression analysis to adjust for confounders. To ensure the reliability of our findings, we analyzed the data after removing the outliers as well as the data after removing the missing values, respectively, yielding consistent outcomes. Furthermore, we executed stratified and subgroup analyses, confirming uniform results across different sex, ACR, BMI, and history of CVD. However, patients aged ≥ 60y, with eGFR < 60 ml/min/1.73m^2^, and diabetes, exhibited a more pronounced trend of increased cardiovascular mortality risk with diminished energy intake. While yielding intriguing results, necessitates further prospective investigations for robust evidence in this study. On the basis of the results, a simple estimation method of 3 and 5-year cardiovascular survival was constructed by nomogram for the special group of patients with CKD on a LPD. This method offers an accessible, efficacious prediction tool, readily applicable and generalizable for CKD patient on a LPD.

Despite its insights, this study bears limitations. The reliance on 24-hour dietary recall may not mirror long-term intake accurately. We didn’t control all therapeutic interventions, potentially skewing results. Lastly, findings apply solely to CKD patients on a LPD, not predicting cardiovascular mortality risk in CKD patients on a non-LPD.

Conclusively, our research underscores the dietary state of CKD patients, revealing a notably deficient energy intake in those adhering to a LPD, independently escalating cardiovascular mortality risk. Such discoveries advocate for a moderate elevation in energy consumption among CKD patients on a LPD.

### Electronic supplementary material

Below is the link to the electronic supplementary material.

Supplementary Material 1

Supplementary Material 2

## Data Availability

Some or all datasets generated during and/or analyzed during the current study are not publicly available but are available from the corresponding author on reasonable request.

## Abbreviations

LPD: Low protein diets
VLPD: Very low protein diets
MNT: Medical nutrition therapy
ESRD: End-stage renal disease
BMI: Body mass index
ACR: Albumin-creatinine ratio
e-GFR: Estimated glomerular filtration rate
REE: Resting energy expenditure
RCS: Restricted cubic spline
RAASi: Renin angiotensin aldosterone system inhibitor
CKD: Chronic kidney disease
CVD: Cardiovascular disease
SE: Standard error
PEW: Protein-energy wasting
