# Supplementary material for: Lower energy intake associated with higher risk of cardiovascular mortality in chronic kidney disease patients on a low-protein diets
Source: Nutr J. 2024 Jul 15;23:75. doi: 10.1186/s12937-024-00980-y (PMC11247864; doi:10.1186/s12937-024-00980-y)
Supplement: Supplementary file 1 — Supplementary Material 1 [file 12937_2024_980_MOESM1_ESM.docx]

**
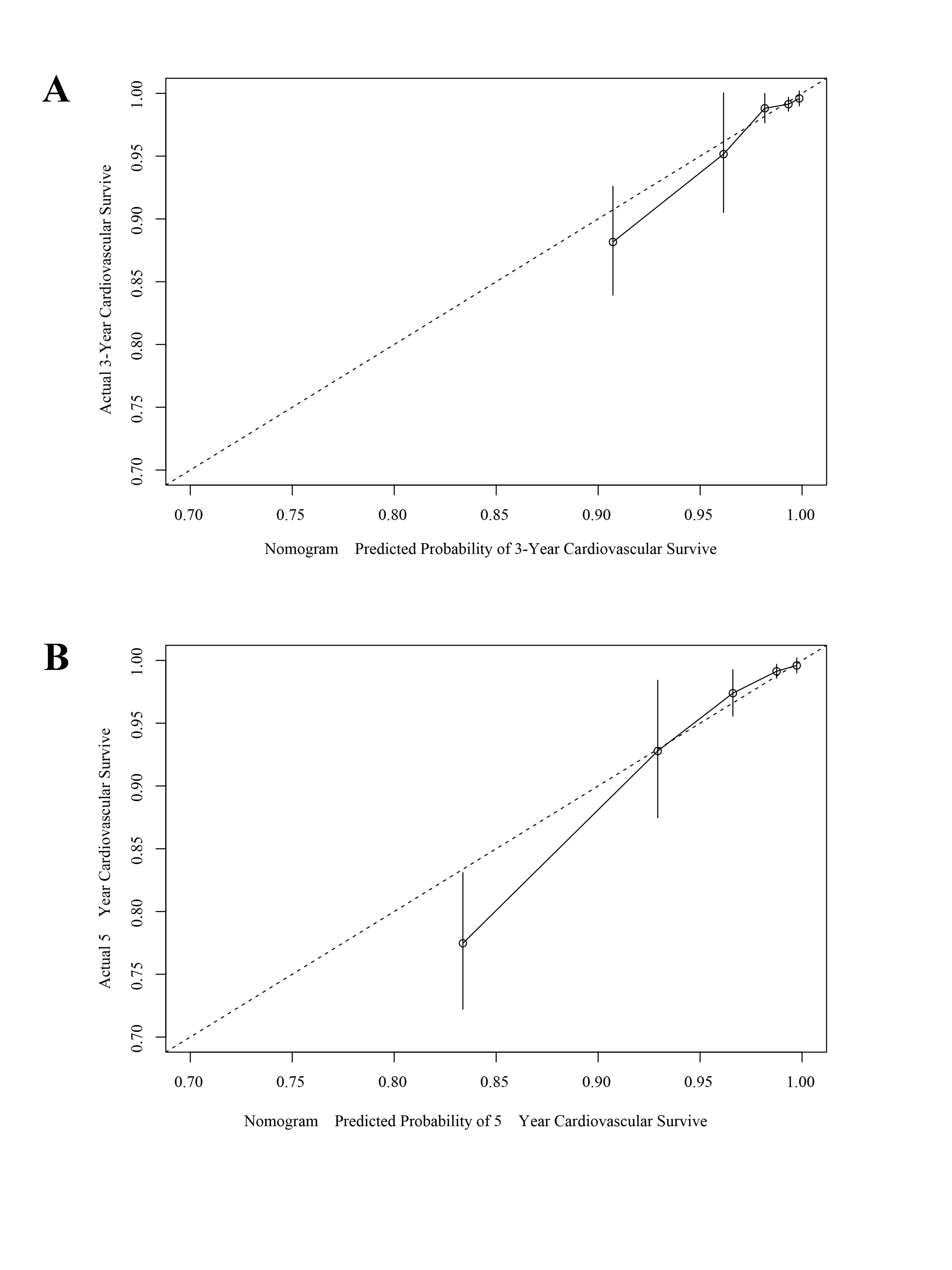
Supplementary -Figure 1.** Calibration curves of the nomogram for predicting renal survival at 3, and 5 years. The nomogram-predicted probability of cardiovascular survival is plotted on the x-axis, and the actual cardiovascular survival is plotted on the y-axis. (A) Calibration curves at 3 years, (B) Calibration curves at 5 years.

**Supplementary -table 1. Definitions/criteria of some diagnoses**

| Variables | Definitions/criteria |
| --- | --- |
| Smoker | Smoking more than 100 cigarettes in previous and now. |
| Alcohol user ^1^ | ≥2 drinks per day for females, ≥3 drinks per day for males, or binge drinking ≥2 days per month.  Binge drinking (≥4 drinks on the same occasion for females, ≥5 drinks on the same occasion for males) on 5 or more days per month. |
| Hypertension ^2^ | 1. Self-reported hypertension diagnosis, (2) Use of anti-hypertensive medication, (3) Average systolic blood pressure (SBP) > 140 mmHg, (4) Average diastolic blood pressure (DBP) > 90 mmHg, meet any of the above conditions. |
| Anemia ^3^ | ≥120g/L for women (15 years of age and above), ≥130g/L for men (15 years of age and above). |
| Hyperlipidemia | (1) Triglyceridemia ≥ 150 mg/dl; (2) Hypercholesterolemia: a) total cholesterol ≥ 200 mg/dl, b) low-density lipoprotein ≥ 130 mg/dl), c). high-density lipoprotein (< 40 mg/dl, male; < 50 mg/dl, female), meet any of the above conditions; (3) Use of lipid-lowering drugs; meet any of the above conditions. |
| Low-carbohydrate diet | Less than 26% of the calories come from carbohydrate or carbohydrate intake less than 130 g/day ^4^. |
| Low-fat diets | Food where less than 26% of the calories come from carbohydrate or carbohydrate intake less than 130 g/day ^5^. |
| Cardiovascular disease | 1) coronary heart disease, 2) congestive heart failure, 3) heart attack, 4) stroke, 5) angina; meet any of the above conditions. |
| Diabetes mellitus | 1) a doctor has told you that you have diabetes, 2) HbA1c (%) > 6.5, 3) fasting blood glucose (mmol/l) ≥ 7.0, 4) random blood glucose (mmol/l) ≥ 11.1, and 5) two-hour oral glucose tolerance test (OGTT) blood glucose (mmol/l) >= 11.1 ^6^. |

**Supplementary -table 2. Status of nutritional intake in CKD patients with changes over the years**

| Variable |  | Year | | | | | P-value |
| --- | --- | --- | --- | --- | --- | --- | --- |
|  | Total | 2009-2010 | 2011-2012 | 2013-2014 | 2015-2016 | 2017-2018 |  |
| Energy intake (kcal/kg/d) | 24.09(0.26) | 23.59(0.66) | 24.21(0.51) | 24.07(0.61) | 24.68(0.73) | 23.82(0.36) | 0.82 |
| Protein intake (g/kg/d) | 0.92(0.01) | 0.92(0.02) | 0.92(0.03) | 0.93(0.03) | 0.92(0.02) | 0.90(0.03) | 0.94 |
| Carbohydrate (g/kg/d) | 2.90(0.03) | 2.90(0.09) | 3.00(0.06) | 2.87(0.06) | 2.93(0.07) | 2.83(0.06) | 0.32 |
| Fat (g/kg/d) | 0.93(0.01) | 0.90(0.03) | 0.90(0.03) | 0.92(0.03) | 0.96(0.03) | 0.96(0.02) | 0.14 |
| Energy intake (kcal/kg/d) |  |  |  |  |  |  | 0.82 |
| <25 | 61.60(0.02) | 65.35(2.09) | 59.57(2.42) | 61.77(1.62) | 59.51(3.19) | 62.49(2.86) |  |
| 25-35 | 23.94(0.01) | 21.95(1.58) | 24.70(2.60) | 22.86(1.79) | 25.94(2.59) | 23.85(2.73) |  |
| ≥35 | 14.46(0.01) | 12.70(1.55) | 15.73(2.32) | 15.37(1.86) | 14.55(1.77) | 13.66(1.47) |  |
| Energy intake greater than the equation-based REE (%) |  |  |  |  |  |  | 0.76 |
| No | 31.57(0.01) | 31.44(1.98) | 32.55(1.95) | 32.53(2.28) | 29.01(2.58) | 32.39(2.66) |  |
| Yes | 68.43(0.02) | 68.56(1.98) | 67.45(1.95) | 67.47(2.28) | 70.99(2.58) | 67.61(2.66) |  |
| LPD (%) |  |  |  |  |  |  | 0.51 |
| No | 53.57(0.02) | 53.96(1.75) | 51.92(2.24) | 50.97(1.46) | 55.53(2.79) | 55.37(3.10) |  |
| Yes | 46.43(0.02) | 46.04(1.75) | 48.08(2.24) | 49.03(1.46) | 44.47(2.79) | 44.63(3.10) |  |
| Very low protein diet (%) |  |  |  |  |  |  | 0.73 |
| No | 73.47(0.03) | 74.24(2.24) | 73.19(2.30) | 71.18(2.36) | 75.97(2.73) | 72.96(3.17) |  |
| Yes | 26.53(0.01) | 25.76(2.24) | 26.81(2.30) | 28.82(2.36) | 24.03(2.73) | 27.04(3.17) |  |
| Low carbohydrate diet (%) |  |  |  |  |  |  | 0.07 |
| No | 97.72(0.03) | 99.04(0.42) | 98.85(0.44) | 97.66(0.65) | 96.47(1.14) | 97.01(0.93) |  |
| Yes | 2.28(0.00) | 0.96(0.42) | 1.15(0.44) | 2.34(0.65) | 3.53(1.14) | 2.99(0.93) |  |
| Low fat diet (%) |  |  |  |  |  |  | 0.002 |
| No | 69.22(0.02) | 67.22(2.88) | 61.85(2.28) | 68.80(2.28) | 71.89(2.62) | 75.03(1.87) |  |
| Yes | 30.78(0.01) | 32.78(2.88) | 38.15(2.28) | 31.20(2.28) | 28.11(2.62) | 24.97(1.87) |  |
| Low fat and carbohydrate diet (%) |  |  |  |  |  |  | 0.03 |
| No | 99.72(0.03) | 99.94(0.05) | 99.95(0.06) | 99.21(0.55) | 99.82(0.11) | 99.76(0.18) |  |
| Yes | 0.28(0.00) | 0.06(0.05) | 0.05(0.06) | 0.79(0.55) | 0.18(0.11) | 0.24(0.18) |  |
| Low LPD and fat and carbohydrate diet (%) |  |  |  |  |  |  | < 0.001 |
| No | 99.77(0.03) | 99.98(0.02) | 99.95(0.06) | 99.21(0.55) | 99.86(0.10) | 99.93(0.07) |  |
| Yes | 0.23(0.00) | 0.02(0.02) | 0.05(0.06) | 0.79(0.55) | 0.14(0.10) | 0.07(0.07) |  |

LPD, low protein diet; REE, resting energy expenditure; CKD, chronic kidney disease.

**Supplementary -table 3. Cox-regression analysis of risk factors for cardiovascular mortality in CKD on a LPD**

| Variables | Unadjusted | | | Model 3 | | |
| --- | --- | --- | --- | --- | --- | --- |
|  | HR | 95%CI | P-value | HR | (95%CI) | P-value |
| Energy intake |  |  |  |  |  |  |
| Tertile 3 | ref | ref |  | ref | ref |  |
| Tertile 2 | 2.54 | (1.45,4.45) | 0.001 | 1.97 | (1.10, 3.52) | 0.02 |
| Tertile 1 | 2.48 | (1.38,4.44) | 0.002 | 2.37 | (1.38, 4.06) | 0.002 |
| Age | 1.09 | (1.06,1.13) | <0.001 | 1.09 | (1.05, 1.12) | <0.001 |
| Sex |  |  |  |  |  |  |
| Female | ref | ref |  | ref | ref |  |
| Male | 1.26 | (0.79,2.02) | 0.33 | 0.98 | (0.54, 1.78) | 0.96 |
| BMI (kg/m^2^) |  |  |  |  |  |  |
| <30 | ref | ref |  | ref | ref |  |
| ≥30 | 0.69 | (0.39,1.22) | 0.20 | 0.61 | (0.35, 1.04) | 0.07 |
| Ethnicity |  |  |  |  |  |  |
| Mexican American | ref | ref |  | ref | ref |  |
| Non-Hispanic Black | 1.28 | (0.60,2.74) | 0.52 | 0.58 | (0.23, 1.48) | 0.26 |
| Non-Hispanic White | 2.03 | (1.21,3.43) | 0.01 | 1.07 | (0.58, 2.01) | 0.82 |
| Other Hispanic | 0.80 | (0.31,2.02) | 0.63 | 0.51 | (0.20, 1.32) | 0.17 |
| Other ethnicity - Including Multi-Racial | 1.10 | (0.20,6.10) | 0.92 | 0.77 | (0.14, 4.22) | 0.76 |
| Alcohol use |  |  |  |  |  |  |
| No | ref | ref |  | ref | ref |  |
| Yes | 0.88 | 0.88(0.53,1.46) | 0.62 | 1.20 | (0.71, 2.02) | 0.50 |
| Smoke |  |  |  |  |  |  |
| No | ref | ref |  | ref | ref |  |
| Yes | 1.16 | 1.16(0.67,2.01) | 0.60 | 1.10 | (0.64, 1.89) | 0.73 |
| e-GFR (ml/min/1.73m2) |  |  |  |  |  |  |
| ≥90 | ref | ref |  | ref | ref |  |
| 60≤e-GFR<90 | 8.87 | (3.59, 21.93) | <0.001 | 1.99 | (0.76, 5.22) | 0.16 |
| 30≤e-GFR<60 | 10.93 | (4.63, 25.81) | <0.001 | 2.61 | (0.87, 7.77) | 0.09 |
| 15≤e-GFR<30 | 16.75 | (5.89, 47.64) | <0.001 | 2.21 | (0.68, 7.20) | 0.19 |
| <15 | 39.41 | (9.37,165.73) | <0.001 | 7.21 | (1.65,31.42) | 0.01 |
| Serum albumin (g/L) |  |  |  |  |  |  |
| <35 | ref | ref |  | ref | ref |  |
| ≥35 | 3.22 | (1.33,7.80) | 0.01 | 2.61 | (0.93, 7.37) | 0.07 |
| ACR (mg/g) |  |  |  |  |  |  |
| <30 | ref | ref |  | ref | ref |  |
| 30≤acr<300 | 0.83 | (0.52,1.32) | 0.43 | 1.86 | (1.00, 3.45) | 0.05 |
| ≥300 | 0.93 | (0.44,1.96) | 0.84 | 1.27 | (0.55, 2.92) | 0.57 |
| Diabetes mellitus |  |  |  |  |  |  |
| No | ref | ref |  | ref | ref |  |
| Yes | 1.92 | (1.36,2.70) | <0.001 | 1.82 | (1.26, 2.62) | 0.001 |
| Hyperlipidemia |  |  |  |  |  |  |
| No | ref | ref |  | ref | ref |  |
| Yes | 1.00 | (0.63,1.59) | 0.99 | 0.75 | (0.45, 1.24) | 0.26 |
| Hypertension |  |  |  |  |  |  |
| No | ref | ref |  | ref | ref |  |
| Yes | 1.83 | (1.12,2.98) | 0.01 | 0.82 | (0.52, 1.31) | 0.41 |
| Anemia |  |  |  |  |  |  |
| No | ref | ref |  | ref | ref |  |
| Yes | 3.73 | (2.12,6.57) | <0.001 | 2.89 | (1.63, 5.09) | <0.001 |
| RAASi |  |  |  |  |  |  |
| No | ref | ref |  | ref | ref |  |
| Yes | 1.00 | (0.64,1.59) | 0.99 | 0.51 | (0.25, 1.01) | 0.05 |

HR, hazard ratio; CI, confidence interval; BMI, body mass index; ACR, albumin-creatinine ratio; e-GFR, estimated glomerular filtration rate; CKD, chronic kidney disease; LPD, low protein diet; RAASi, renin-angiotensin-aldosterone system inhibitor;

**Supplementary -table 4**. **Associations between energy intake and cardiovascular mortality in CKD on a LPD**

| Variables | Unadjusted | | Model 1^a^ | | Model 2^b^ | | Model 3^c^ | |
| --- | --- | --- | --- | --- | --- | --- | --- | --- |
|  | HR (95%CI) | P | HR (95%CI) | P | HR (95%CI) | P | HR (95%CI) | P |
| Tertile 3 | ref |  | ref |  | ref |  | ref |  |
| Tertile 2 | 2.54(1.45,4.45) | 0.001 | 2.35(1.39,3.99) | 0.001 | 2.38(1.36, 4.18) | 0.002 | 1.97(1.10, 3.52) | 0.02 |
| Tertile 1 | 2.48(1.38,4.44) | 0.002 | 2.77(1.59,4.84) | <0.001 | 2.76(1.60, 4.77) | <0.001 | 2.37(1.38, 4.06) | 0.002 |
| P for trend |  | 0.002 |  | <0.001 |  | <0.001 |  | 0.003 |
| Per-SD increment of energy intake | 1.41(1.13,1.76) | 0.002 | 1.49(1.17,1.92) | 0.002 | 1.48(1.18, 1.87) | <0.001 | 1.41(1.12, 1.77) | 0.004 |

Model 1^a^ adjusted for baseline age, sex (‘Male’, ‘Female’), ethnic, BMI (‘<30’, ‘≥30’), alcohol use (‘No’, ‘Yes’), smoke (‘No’, ‘’Yes); Model 2^b^ adjusted for covariates in model 1 plus e-GFR, ACR (‘<30’, ‘30-300’, ‘≥30’); Model 3^c^ adjusted for covariates in model 2 plus serum albumin (‘<35’, ‘≥35’), hypertension (‘Yes’ or ‘No’), anemia (‘Yes’ or ‘No’), diabetes (‘Yes’ or ‘No’), hyperlipidemia (‘Yes’ or ‘No’), RASSi use (‘Yes’ or ‘No’); HR, hazard ratio; CI, confidence interval; BMI, body mass index; ACR, albumin-creatinine ratio; e-GFR, estimated glomerular filtration rate; RAASi, renin-angiotensin-aldosterone system inhibitor; CKD, chronic kidney disease.

**Supplementary -table 5**. **Stratified analysis of the effect of energy intake and the cardiovascular mortality in patients with CKD on a LPD**

| Variables | HR (95% CI) | | | P for trend | P for interaction |
| --- | --- | --- | --- | --- | --- |
|  | Tertile 3 | Tertile 2 | Tertile 1 |  |  |
| Age (years) |  |  |  |  | 0.02 |
| <60 | ref | 0.38(0.06, 2.19) | 3.12(0.53, 18.55) | 0.17 |  |
| ≥60 | ref | 2.22(1.24,3.99) | 1.85(1.05,3.26) | 0.05 |  |
| Sex (%) |  |  |  |  | 0.95 |
| Female | ref | 2.03(0.91,4.52) | 2.45(1.13,5.32) | 0.03 |  |
| Male | ref | 1.90(0.86,4.21) | 1.75(0.70,4.38) | 0.28 |  |
| BMI (kg/m^2^) |  |  |  |  | 0.91 |
| <30 | ref | 2.09( 0.79, 5.55) | 1.84( 0.55, 6.17) | 0.25 |  |
| ≥30 | ref | 2.54(1.26,5.12) | 2.74(1.44,5.21) | 0.002 |  |
| ACR (mg/g) |  |  |  |  | 0.09 |
| <30 | ref | 3.30(1.51,7.18) | 1.82(0.64,5.17) | 0.48 |  |
| 30-300 | ref | 1.73(0.80,3.73) | 2.28(0.78,6.67) | 0.14 |  |
| ≥300 | ref | 0.32(0.04,2.35) | 1.09(0.26,4.61) | 0.57 |  |
| e-GFR (ml/min/1.73^2^) |  |  |  |  | <0.001 |
| <60 | ref | 0.53(0.21,1.36) | 1.57(0.75,3.31) | 0.25 |  |
| ≥60 | ref | 3.79(1.99,7.21) | 3.14(1.38,7.13) | 0.02 |  |
| Diabetes |  |  |  |  | 0.03 |
| No | ref | 1.20(0.44,3.27) | 1.69(0.83,3.44) | 0.08 |  |
| Yes | ref | 3.04(1.56, 5.89) | 1.72(0.64, 4.59) | 0.19 |  |
| CVD |  |  |  |  | 0.32 |
| No | ref | 1.44(0.63,3.30) | 2.39(1.28,4.49) | 0.01 |  |
| Yes | ref | 2.64(1.23,5.69) | 2.50(1.06,5.89) | 0.05 |  |

Adjusted for baseline age, sex, ethnicity,BMI, e-GFR, ACR, e-GFR; HR, hazard ratio; CI, confidence interval; ACR, albumin-creatinine ratio; e-GFR, estimated glomerular filtration rate; CKD, chronic kidney disease; CVD, Cardiovascular Disease

**Supplementary -table 6. Subgroup analysis of the effect of energy intake and the cardiovascular mortality in patients with CKD on a LPD**

| Variables | Tertile 3 | Tertile 2 | | Tertile 1 | |
| --- | --- | --- | --- | --- | --- |
|  |  | HR (95% CI) | P | HR (95% CI) | P |
| Age (years) |  |  |  |  |  |
| <60 | ref | 2.97(1.69, 5.23) | <0.001 | 2.89(1.79, 4.65) | <0.001 |
| ≥60 | ref | 1.04(0.77, 1.41) | 0.8 | 1.01(0.71, 1.42) | 0.96 |
| e-GFR |  |  |  |  |  |
| <45 | ref | 1.46(1.01,2.11) | 0.04 | 1.73(1.21,2.47) | 0.003 |
| ≥45 | ref | 0.97(0.79, 1.19) | 0.77 | 0.94(0.74, 1.19) | 0.60 |
| Diabetes |  |  |  |  |  |
| No | ref | 1.07(0.77,1.47) | 0.69 | 1.36(0.95,1.94) | 0.09 |
| Yes | ref | 1.78(1.22,2.59) | 0.003 | 1.89(1.29,2.78) | 0.001 |

Model 1^a^ adjusted for baseline age, sex (‘Male’, ‘Female’), ethnic, BMI (‘<30’, ‘≥30’), alcohol use (‘No’, ‘Yes’), smoke (‘No’, ‘’Yes), e-GFR, ACR (‘<30’, ‘30-300’, ‘≥30’), albumin (‘<35’, ‘≥35’), hypertension (‘Yes’ or ‘No’), anemia (‘Yes’ or ‘No’), diabetes (‘Yes’ or ‘No’), hyperlipidemia (‘Yes’ or ‘No’), RASSi use (‘Yes’ or ‘No’); HR, hazard ratio; CI, Confidence interval; BMI, body mass index; ACR, albumin-creatinine ratio; e-GFR, estimated glomerular filtration rate; RAASi, renin-angiotensin-aldosterone system inhibitor; CKD, chronic kidney disease.

**Supplementary -table 7. Relationship between energy intake and cardiovascular mortality in CKD patients on LPD based on data from deleted outliers**

| Variables | Unadjusted | | Model 1^a^ | | Model 2^b^ | | Model 3^c^ | |
| --- | --- | --- | --- | --- | --- | --- | --- | --- |
|  | HR (95%CI) | P | HR (95%CI) | P | HR (95%CI) | P | HR (95%CI) | P |
| Tertile 3 | ref |  | ref |  | ref |  | ref |  |
| Tertile 2 | 2.53(1.36,4.70) | 0.003 | 2.31(1.32,4.04) | 0.003 | 2.46(1.36, 4.44) | 0.003 | 2.19(1.21, 3.95) | 0.01 |
| Tertile 1 | 1.92(1.18,3.13) | 0.01 | 2.07(1.26,3.42) | 0.004 | 2.14(1.29, 3.55) | 0.003 | 1.81(1.08, 3.04) | 0.03 |
| P for trend |  | 0.01 |  | 0.004 |  | 0.002 |  | 0.03 |
| Per-SD increment of energy intake | 1.36(1.14,1.63) | <0.001 | 1.41(1.16,1.72) | <0.001 | 1.43(1.18, 1.74) | <0.001 | 1.33(1.09, 1.63) | 0.005 |

Model 1^a^ adjusted for baseline age, sex (‘Male’, ‘Female’), ethnic, BMI (‘<30’, ‘≥30’), alcohol use (‘No’, ‘Yes’), smoke (‘No’, ‘’Yes); Model 2^b^ adjusted for covariates in model 1 plus e-GFR, ACR (‘<30’, ‘30-300’, ‘≥30’); Model 3^c^ adjusted for covariates in model 2 plus serum albumin (‘<35’, ‘≥35’), hypertension (‘Yes’ or ‘No’), anemia (‘Yes’ or ‘No’), diabetes (‘Yes’ or ‘No’), hyperlipidemia (‘Yes’ or ‘No’), RASSi use (‘Yes’ or ‘No’); HR, hazard ratio; CI, confidence interval; BMI, body mass index; ACR, albumin-creatinine ratio; e-GFR, estimated glomerular filtration rate; RAASi, renin-angiotensin-aldosterone system inhibitor; CKD, chronic kidney disease.

**Supplementary -table 8. Relationship between energy intake and cardiovascular mortality in CKD patients on LPD based on data missing values deleted**

| Variables | Unadjusted | | Model 1^a^ | | Model 2^b^ | | Model 3^c^ | |
| --- | --- | --- | --- | --- | --- | --- | --- | --- |
|  | HR (95%CI) | P | HR (95%CI) | P | HR (95%CI) | P | HR (95%CI) | P |
| Tertile 3 | ref |  | ref |  | ref |  | ref |  |
| Tertile 2 | 2.54(1.45,4.45) | 0.001 | 2.48(1.42, 4.31) | 0.001 | 2.44(1.42, 4.31) | 0.004 | 2.02(1.06, 3.86) | 0.03 |
| Tertile 1 | 2.48(1.38,4.44) | <0.001 | 2.83(1.59, 5.05) | <0.001 | 2.83(1.59, 5.05) | <0.001 | 2.25(1.33, 3.81) | 0.003 |
| P for trend |  | <0.001 |  | <0.001 |  | <0.001 |  | 0.003 |
| Per-SD increment of energy intake | 1.41(1.13,1.76) | 0.002 | 1.51(1.17,1.96) | 0.002 | 1.51(1.17, 1.94) | 0.002 | 1.42(1.11, 1.81) | 0.005 |

Model 1^a^ adjusted for baseline age, sex (‘Male’, ‘Female’), ethnic, BMI (‘<30’, ‘≥30’), alcohol use (‘No’, ‘Yes’), smoke (‘No’, ‘’Yes); Model 2^b^ adjusted for covariates in model 1 plus e-GFR, ACR (‘<30’, ‘30-300’, ‘≥30’); Model 3^c^ adjusted for covariates in model 2 plus serum albumin (‘<35’, ‘≥35’), hypertension (‘Yes’ or ‘No’), anemia (‘Yes’ or ‘No’), diabetes (‘Yes’ or ‘No’), hyperlipidemia (‘Yes’ or ‘No’), RASSi use (‘Yes’ or ‘No’); HR, hazard ratio; CI, confidence interval; BMI, body mass index; ACR, albumin-creatinine ratio; e-GFR, estimated glomerular filtration rate; RAASi, renin-angiotensin-aldosterone system inhibitor; CKD, chronic kidney disease.

**Supplementary -table 9**. **Associations between energy intake and cardiovascular mortality in CKD on a LPD as determined by dichotomous grouping of energy intake**

| Variables | Unadjusted | | Model 1^a^ | | Model 2^b^ | | Model 3^c^ | |
| --- | --- | --- | --- | --- | --- | --- | --- | --- |
|  | HR (95%CI) | P | HR (95%CI) | P | HR (95%CI) | P | HR (95%CI) | P |
| Q 2 | ref |  | ref |  | ref |  | ref |  |
| Q 1 | 1.86(1.17,2.95) | 0.01 | 2.01(1.26,3.19) | 0.003 | 2.07(1.32, 3.26) | 0.002 | 1.91(1.20, 3.05) | 0.01 |

Model 1^a^ adjusted for baseline age, sex (‘Male’, ‘Female’), ethnic, BMI (‘<30’, ‘≥30’), alcohol use (‘No’, ‘Yes’), smoke (‘No’, ‘’Yes); Model 2^b^ adjusted for covariates in model 1 plus e-GFR, ACR (‘<30’, ‘30-300’, ‘≥30’); Model 3^c^ adjusted for covariates in model 2 plus serum albumin (‘<35’, ‘≥35’), hypertension (‘Yes’ or ‘No’), anemia (‘Yes’ or ‘No’), diabetes (‘Yes’ or ‘No’), hyperlipidemia (‘Yes’ or ‘No’), RASSi use (‘Yes’ or ‘No’); HR, hazard ratio; CI, confidence interval; BMI, body mass index; ACR, albumin-creatinine ratio; e-GFR, estimated glomerular filtration rate; RAASi, renin-angiotensin-aldosterone system inhibitor; CKD, chronic kidney disease.

**Supplementary -table 10**. **Associations between energy intake and cardiovascular mortality in CKD on a LPD as determined by quartile grouping of energy intake**

| Variables | Unadjusted | | Model 1^a^ | | Model 2^b^ | | Model 3^c^ | |
| --- | --- | --- | --- | --- | --- | --- | --- | --- |
|  | HR (95%CI) | P | HR (95%CI) | P | HR (95%CI) | P | HR (95%CI) | P |
| Q 4 | ref |  | ref |  | ref |  | ref |  |
| Q 3 | 1.49(0.76,2.90) | 0.24 | 1.37(0.75,2.48) | 0.3 | 1.28(0.70, 2.34) | 0.42 | 1.16(0.63, 2.15) | 0.63 |
| Q 2 | 2.09(1.04,4.21) | 0.04 | 2.02(1.04,3.94) | 0.04 | 2.07(1.06, 4.03) | 0.03 | 1.89(0.96, 3.70) | 0.06 |
| Q 1 | 2.49(1.34,4.63) | 0.004 | 2.83(1.55,5.17) | <0.001 | 2.73(1.51, 4.92) | <0.001 | 2.30(1.30, 4.07) | 0.004 |
| P for trend |  | 0.003 |  | <0.001 |  | <0.001 |  | 0.003 |

Model 1^a^ adjusted for baseline age, sex (‘Male’, ‘Female’), ethnic, BMI (‘<30’, ‘≥30’), alcohol use (‘No’, ‘Yes’), smoke (‘No’, ‘’Yes); Model 2^b^ adjusted for covariates in model 1 plus e-GFR, ACR (‘<30’, ‘30-300’, ‘≥30’); Model 3^c^ adjusted for covariates in model 2 plus serum albumin (‘<35’, ‘≥35’), hypertension (‘Yes’ or ‘No’), anemia (‘Yes’ or ‘No’), diabetes (‘Yes’ or ‘No’), hyperlipidemia (‘Yes’ or ‘No’), RASSi use (‘Yes’ or ‘No’); HR, hazard ratio; CI, confidence interval; BMI, body mass index; ACR, albumin-creatinine ratio; e-GFR, estimated glomerular filtration rate; RAASi, renin-angiotensin-aldosterone system inhibitor; CKD, chronic kidney disease.

**Supplementary -table 11**. **Percentage of patients with Digestive System Tumors, COPD, and depressive disorders with different energy intakes**

| Variable | Total  (N=2021, unweighted) | Tertile 1  (≤13.46 kcal/kg/d)  (n=674, unweighted) | Tertile 2  (13.46-18.58 kcal/kg/d)  (n=673, unweighted) | Tertile 3  (>18.58 cal/kg/d)  (n=674, unweighted) | P-value |
| --- | --- | --- | --- | --- | --- |
| Energy intake (kcal/kg/d) | 16.74(0.20) | 10.15(0.13) | 16.00(0.08) | 23.72(0.26) | < 0.001 |
| Digestive System Tumors (%) |  |  |  |  | 0.13 |
| No | 77.82(0.03) | 98.63(0.51) | 97.49(0.85) | 99.07(0.36) |  |
| Yes | 1.26(0.00) | 1.37(0.51) | 2.51(0.85) | 0.93(0.36) |  |
| COPD |  |  |  |  | 0.86 |
| No | 91.73(0.04) | 91.22(1.30) | 92.17(1.20) | 91.78(1.30) |  |
| Yes | 8.27(0.01) | 8.78(1.30) | 7.83(1.20) | 8.22(1.30) |  |
| Depression |  |  |  |  | 0.62 |
| No | 66.79(0.03) | 69.59(2.25) | 72.41(2.63) | 69.27(2.74) |  |
| Yes | 28.07(0.02) | 30.41(2.25) | 27.59(2.63) | 30.73(2.74) |  |

**Supplementary -table 12**. **Associations between energy intake and cardiovascular mortality in CKD on a LPD**

| Variables | Unadjusted | | Model 1^a^ | | Model 2^b^ | | Model 3^c^ | |
| --- | --- | --- | --- | --- | --- | --- | --- | --- |
|  | HR (95%CI) | P | HR (95%CI) | P | HR (95%CI) | P | HR (95%CI) | P |
| Q 3 (n=432)  （Energy intake＞18.75 | ref |  | ref |  | ref |  | ref |  |
| Q 2 (n=432)  （13.61<Energy intake≤18.75） | 2.67(1.35,5.26) | 0.005 | 2.25(1.14,4.47) | 0.02 | 2.28(1.08, 4.82) | 0.03 | 2.22(0.99, 4.96) | 0.05 |
| Q 1 (n=432)  （Energy intake<13.61） | 2.92(1.44,5.92) | 0.003 | 3.20(1.62,6.32) | <0.001 | 3.27(1.63, 6.57) | <0.001 | 2.56(1.34, 4.91) | 0.004 |
| P for trend |  | 0.01 |  | 0.003 |  | 0.002 |  | 0.01 |

Model 1^a^ adjusted for baseline age, sex (‘Male’, ‘Female’), ethnic, BMI (‘<30’, ‘≥30’), alcohol use (‘No’, ‘Yes’), smoke (‘No’, ‘’Yes); Model 2^b^ adjusted for covariates in model 1 plus e-GFR, ACR (‘<30’, ‘30-300’, ‘≥30’); Model 3^c^ adjusted for covariates in model 2 plus serum albumin (‘<35’, ‘≥35’), hypertension (‘Yes’ or ‘No’), anemia (‘Yes’ or ‘No’), diabetes (‘Yes’ or ‘No’), hyperlipidemia (‘Yes’ or ‘No’), RASSi use (‘Yes’ or ‘No’); HR, hazard ratio; CI, confidence interval; BMI, body mass index; ACR, albumin-creatinine ratio; e-GFR, estimated glomerular filtration rate; RAASi, renin-angiotensin-aldosterone system inhibitor; CKD, chronic kidney disease.

**References**

**1.** Rattan P, Penrice DD, Ahn JC, et al. Inverse Association of Telomere Length With Liver Disease and Mortality in the US Population. *Hepatol Commun.* Feb 2022;6(2):399-410.

**2.** Whelton PK, Carey RM, Aronow WS, et al. 2017 ACC/AHA/AAPA/ABC/ACPM/AGS/APhA/ASH/ASPC/NMA/PCNA Guideline for the Prevention, Detection, Evaluation, and Management of High Blood Pressure in Adults: A Report of the American College of Cardiology/American Heart Association Task Force on Clinical Practice Guidelines. *J Am Coll Cardiol.* May 15 2018;71(19):e127-e248.

**3.** WHO. Haemoglobin concentrations for the diagnosis of anaemia and assessment of severity. Vitamin and Mineral Nutrition Information System. Geneva, World Health Organization, 2011 (WHO/NMH/NHD/MNM/11.1).

**4.** Wang J, Zhang J, Lin X, et al. DCA-TGR5 signaling activation alleviates inflammatory response and improves cardiac function in myocardial infarction. *J Mol Cell Cardiol.* Feb 2021;151:3-14.

**5.** Hu X, Yan J, Huang L, et al. INT-777 attenuates NLRP3-ASC inflammasome-mediated neuroinflammation via TGR5/cAMP/PKA signaling pathway after subarachnoid hemorrhage in rats. *Brain Behav Immun.* Jan 2021;91:587-600.

**6.** Chittka D, Banas B, Lennartz L, et al. Long-term expression of glomerular genes in diabetic nephropathy. *Nephrol Dial Transplant.* Sep 1 2018;33(9):1533-1544.
