# Supplementary figures and images for: Lower energy intake associated with higher risk of cardiovascular mortality in chronic kidney disease patients on a low-protein diets
Source: Nutr J. 2024 Jul 15;23:75. doi: 10.1186/s12937-024-00980-y (PMC11247864; doi:10.1186/s12937-024-00980-y)

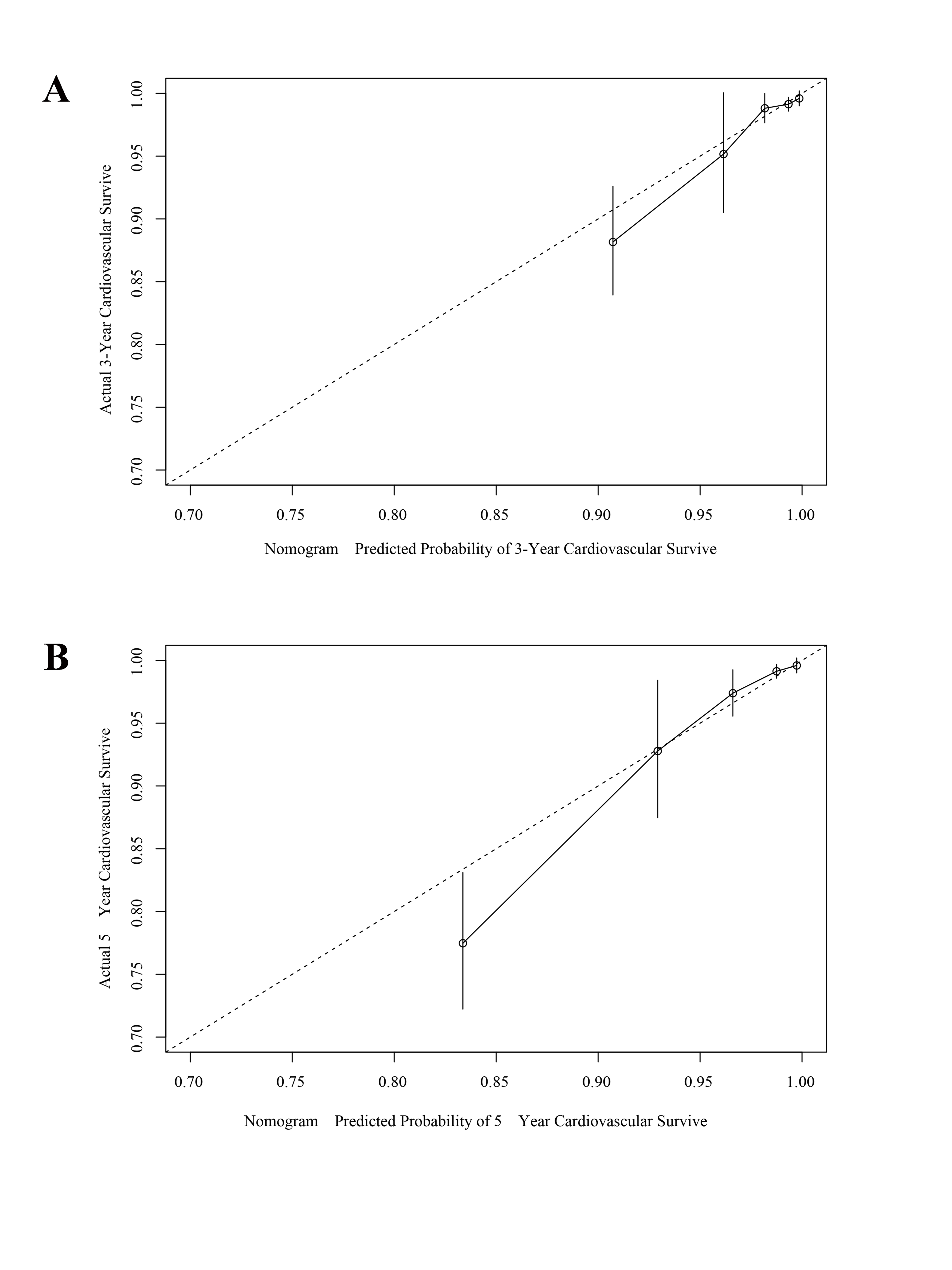

Supplement: Supplementary file 2 — Supplementary Material 2 [file 12937_2024_980_MOESM2_ESM.tif]
